# Health Care Utilization Among Pregnant Women With Pre-existing Kidney Disease: A Retrospective Cohort Analysis

**DOI:** 10.1016/j.xkme.2025.101147

**Published:** 2025-10-14

**Authors:** Sonali Gupta, Aastha Vasa, Katherine Rizzolo, Lilia Cervantes, Belinda Jim, Kavita Vani, Diana Wolfe, Jaya Isaac, Kimberly Reidy, Ladan Golestaneh

**Affiliations:** 1 Division of Nephrology, Department of Medicine, Albert Einstein College of Medicine/Montefiore Medical Center, Bronx, NY; 2 Division of Nephrology, Department of Medicine, Boston University Chobanian and Avedisian School of Medicine, Boston, MA; 3 Division of Hospital Medicine, Division of General Internal Medicine, Department of Medicine, University of Colorado, School of Medicine, Aurora, CO; 4 Division of Nephrology, Department of Medicine, Albert Einstein College of Medicine, Jacobi Medical Center, Bronx, NY; 5 Division of Maternal and Fetal Medicine, Department of Obstetrics and Gynecology, Montefiore Medical Center, Albert Einstein College of Medicine, Bronx, NY; 6 Division of Pediatric Nephrology, Department of Pediatrics, Montefiore Medical Center/Albert Einstein College of Medicine, Bronx, NY; 7 Section of Nephrology, Department of Medicine, Yale University School of Medicine, New Haven, CT

**Keywords:** Pregnancy, chronic kidney disease, proteinuria, prepregnancy CKD

## Abstract

**Rationale & Objective:**

Pregnant women with prepregnancy chronic kidney disease (CKD) are underdiagnosed and experience adverse outcomes. Limited research exists on peripregnancy health care utilization by women with evidence of CKD.

**Study Design:**

Retrospective cohort analysis.

**Setting & Participants:**

Women with laboratory signs of prepregnancy CKD who had a pregnancy event between January 1, 2016, and March 1, 2023, at Montefiore Health System in Bronx, NY, were included.

**Exposure & Outcomes:**

We investigated rates of CKD diagnosis, adverse maternal and fetal outcomes, and peripregnancy health care utilization among a sample of women with evidence of prepregnancy CKD.

**Analytical Approach:**

We tested the proportion of women with laboratory signs of CKD who had a diagnosis code for CKD entered on their problem list. We also examined the association of different eGFR/proteinuria cutoffs among the cohort with (1) adverse pregnancy outcomes, (2) postpregnancy CKD prevalence, and (3) peripregnancy health utilization.

**Results:**

Of the 247 pregnant women who met criteria for prepregnancy CKD by estimated glomerular filtration rate (eGFR) or proteinuria, 8.1% had spontaneous abortion, 10.9% had missed abortion, 12.9% had CKD listed in problem list. Out of 80.9% of pregnancy that reached fetal viability, 12.5% had early preterm delivery, 22.6% developed gestational hypertension, and 20.0% of their infants required neonatal intensive care unit admission, with worse outcomes seen in those with severe CKD (defined as eGFR < 45 mL/min/1.73 m^2^ or proteinuria > 1,000 mg/g). Among women with CKD, 27.6% were seen by primary care within 1 year before pregnancy while less than 0.1% were ever seen by a nephrologist. Among women with severe CKD, only 40% were seen by nephrology during pregnancy, and less than 50% had a nephrology appointment arranged at discharge.

**Limitations:**

Retrospective analysis of a single-center data source.

**Conclusions:**

Pregnant women with CKD were frequently underdiagnosed and not sufficiently integrated into care before, during, or after pregnancy.

## Plain-Language Summary

Pregnant women with prepregnancy chronic kidney disease (CKD) are often underdiagnosed. This study looked at medical records of women who had signs of CKD before getting pregnant and had a pregnancy event between 2016 and 2023. They studied how much these women used health care services. The study found that out of 247 pregnant women with prepregnancy CKD, only about a quarter saw their primary doctor in the year before pregnancy. Less than 0.1% ever saw a kidney specialist. Even for women with severe CKD, only 40% were seen by nephrology during pregnancy, and less than 50% had a nephrology appointment arranged at discharge. In short, pregnant women with severe CKD often go undiagnosed and do not receive adequate care.

Chronic kidney disease (CKD) is a leading cause of mortality worldwide.^1^^,^^2^ Women of reproductive age increasingly have CKD; this is important because pregnancy in women with CKD carries additional risk for acute kidney injury and is a risk factor of maternal complications of pregnancy, including death,^3^, ^4^, ^5^ and adverse fetal outcomes.^6^, ^7^, ^8^, ^9^, ^10^, ^11^ Early intervention by a nephrologist and multidisciplinary care team, which includes high-risk obstetrics, may reduce adverse outcomes.^12^, ^13^, ^14^ There is no defined prepregnancy estimated glomerular filtration rate (eGFR) threshold below which adverse outcomes are expected, limiting the ability to promote quality standards for heightened and continued surveillance through pregnancy. One study showed a marked increase in risk for maternal and fetal morbidity when eGFR dropped below 60 mL/min/1.73 m^2^ (Kidney Disease: Improving Global Outcomes [KDIGO] CKD G3a) and suggested increasing eGFR thresholds for recommended referral to nephrologist.^9^ Further, in pregnant women who show signs of early CKD, the rate at which it is diagnosed and appropriately followed by providers is likely low but has not been quantified. To make matters worse, stark racial and ethnic disparities exist in maternal health outcomes, with African American women at 3 times and Latina women at 1.25 times higher risk of pregnancy-related mortality than White women, which was partially attributed to lack of access to nephrology care postpartum.^15^ Access to care is important as cardiovascular mortality increases with a reduction in eGFR < 75 mL/min/1.73 m^2^ in young adults. In the Coronary Artery Risk Development In Young Adults study, a mildly lower eGFR of 60-75 mL/min/1.73 m^2^ was associated with a higher left ventricular mass index.^16^, ^17^, ^18^, ^19^, ^20^

In this study we identified and followed a cohort of pregnant women with biochemical signs of CKD, with eGFR <75 mL/min/1.73 m^2^ and/or quantified proteinuria ≥ 200 mg/g, and examined the following: (1) the proportion who had CKD diagnoses entered into their problem list by a clinician, (2) the association of different prepregnancy eGFR and proteinuria thresholds with risk of complications and post-partum kidney disease, and (3) patterns of health care utilization before and after pregnancy. In so doing, we hope to raise awareness of the need for more stringent clinical oversight during and after pregnancy in women with early signs of CKD and to better understand the barriers they may face in accessing postpartum care.

## Methods

### Study Population

We performed a retrospective analysis of women with laboratory signs of prepregnancy CKD who had a pregnancy event between January 1, 2016, and early March 1, 2023, at Montefiore Health System (MHS) in Bronx, NY. To reduce sampling bias (because women travel from outside of the Bronx to MHS for obstetrics care), we included only individuals who were engaged in care with MHS, as defined by a cohort defining prepregnancy clinic visit, and who had signs of CKD, with blood and/or urine testing 6 months to 3 years before pregnancy (Table S1). This inclusion also allowed us to reduce differential bias introduced through those who had no prior laboratory testing before pregnancy as being falsely labeled as having normal kidney function. Because of hyperfiltration during the first 2 trimesters of pregnancy potentially conceal low eGFR and potential laboratory evidence of CKD, we excluded laboratory results from first or second trimester by requiring that the look back period start at 6 months before pregnancy event. We also excluded women with pre-eclampsia in their previous pregnancy as a source of confounding in the definition of prepregnancy CKD. This study was approved by the Einstein-Montefiore Institutional Review Board (IRB# 2022-14427).

Prepregnancy CKD was defined using elements of the KDIGO criteria at an eGFR cutoff of 75 mL/min/1.73 m^2^ (calculated using the CKD-EPI [Chronic Kidney Disease Epidemiology Collaboration] 2021 equation)^21^^,^^22^ or most recent urine total protein or albumin to creatinine ratio >200 mg/g.^23^^,^^24^ According to the KDIGO nomenclature for CKD, the cutoff for CKD G2 is 60-89 mL/min/m^2^. We selected a higher eGFR threshold than 60 mL/min/1.73 m^2^ because it is not a sensitive diagnostic threshold for young women of reproductive age. Among younger persons, mortality is increased at GFR < 75 mL/min/1.73 m^2^, lending support to a higher cutoff used for young women.^17^, ^18^, ^19^, ^20^ Subsequent eGFR values were calculated based on the maximum creatinine during pregnancy, the latest of creatinine values within 1 year after pregnancy, and the latest of creatinine values reported between 1 and 3 years after pregnancy. We chose a quantified proteinuria (inclusive of albuminuria) level of >200 mg/g, favoring a more conservative definition to improve specificity in which either quantified spot urine albumin and/or urine protein levels were used. Only the first pregnancy (among those who had multiple pregnancies during the study period) was included for analysis. Patients were excluded if there was a diagnosis of previous pre-eclampsia. For descriptions of fetal outcomes, we only included pregnancies that reached viability.

### Data Source

We extracted data using Clinical Looking Glass, a database building tool that pulls claims, and clinical and demographic data from the electronic health record at MHS.^25^, ^26^, ^27^, ^28^ We conducted a manual chart review between November 2023 and February 2024 to obtain supplemental individual (as opposed to geocoded) level data on sociodemographic variables, comorbid conditions, health utilization before and after pregnancy, and outcomes during and after pregnancy.

### Outcomes

We calculated the proportion of women with laboratory evidence of prepregnancy CKD who had CKD documented in their electronic health record problem list. To understand pre- and postpartum health care access, we evaluated the number of visits scheduled and attended with a primary care provider or nephrologist at MHS. In the postpartum period, we assessed appointments within 1 year postpartum and beyond 1 year and up to 3 years postpartum. We also calculated the proportion of the cohort with renin-angiotensin aldosterone inhibitors (RAASi) prescribed postpartum.

Maternal health outcomes included type of delivery, gestational age at delivery, pre-eclampsia, and gestational hypertension. Adverse fetal outcomes included preterm birth and low birth weight in those who carried pregnancies to the point at which the fetus was viable. The criterion for early preterm birth was gestational age at birth of less than 34 weeks. The diagnostic criteria for low birth weight (LBW) and very LBW were neonatal birth weights of <2,500 g and <1,500 g, respectively.

#### Exploratory Outcomes

To better understand eGFR and proteinuria thresholds predictive of peri- and postpartum CKD, we defined the following subgroups of CKD: (1) prepregnancy moderate CKD (G3a and /or A2) (eGFR 45-59 mL/min/1.73 m^2^ and/or urine protein or albumin to creatinine ratio > 500 mg/g and ≤ 1,000 mg/g) or (2) prepregnancy severe CKD (G3b and severe and/or A3) (eGFR <45 mL/min/1.73 m^2^ or urine protein or albumin to creatinine ratio > 1,000 mg/g). We compared outcomes to those meeting criteria for CKD G2 and/or A1 (eGFR 60-75 mL/min/1.73 m^2^ and/or urine protein or albumin to creatinine ratio >200 mg/g and ≤500 mg/g). We also examined the course of peri- and postpregnancy kidney outcomes for women with above eGFR and quantified proteinuria thresholds, separately.

### Covariates

Sociodemographic variables including age, race, ethnicity, preferred language, insurance, marital status, and income at the census tract level were extracted from Clinical Looking Glass and chart review. Race and ethnicity were self-reported. Preferred language was captured as English, Spanish, or other language. Insurance status included Medicaid/managed Medicaid, Medicare/managed Medicare, commercial insurance, union, or other type of insurance. Marital status included those who were married/cohabitating and unmarried/single. Income was calculated based on median income of households in census tract of patients’ residential addresses and standardized and centered at NYS state mean (mean = 0). We included comorbid conditions often associated with CKD based on claims entered for obesity, diabetes, and hypertension within 5 years of prepregnancy.

### Statistical Analysis

Baseline demographic, clinical, and laboratory characteristics were presented using descriptive statistics including mean (SD) or median (interquartile range [IQR]) for continuous variables and counts and proportions for categorical variables. One way analysis of variance and Kruskal–Wallis (depending on distribution) tests were used for comparing continuous or ordinal outcomes across categories of CKD thresholds. In addition, χ^2^ test were used for assessing associations between categorical variables and outcomes. Data analyses were performed using STATA version 17.0 (Stata Corp, College Station, TX). Statistical significance was defined at *α* ≤ 0.05.

## Results

### Patient Characteristics

**Figure 1:**
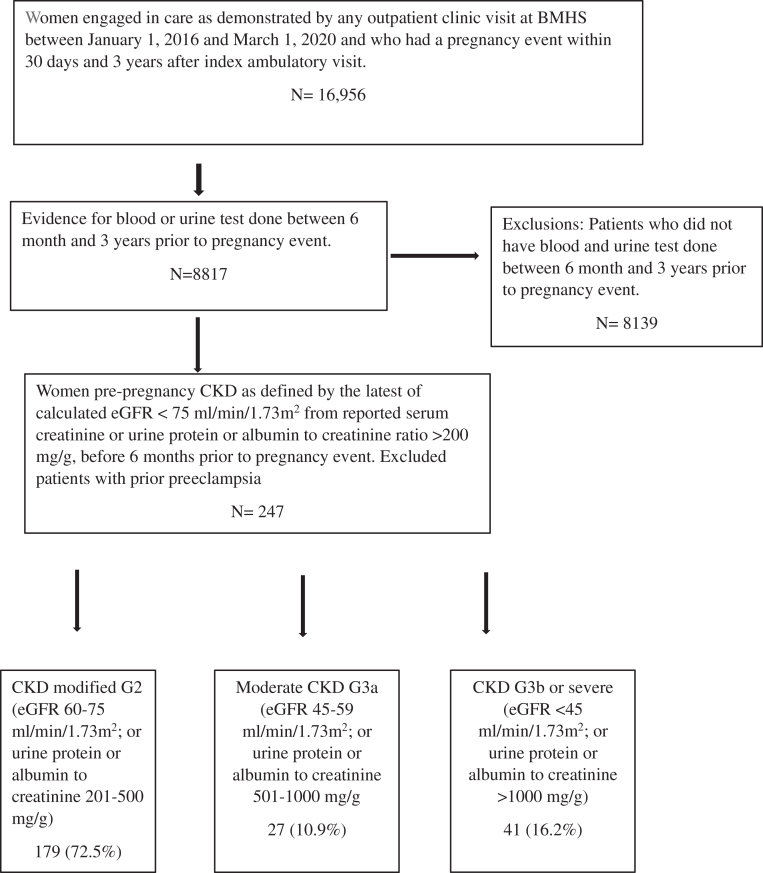
Study population.

Of 16,956 women who had a pregnancy event at MHS, we excluded 8,139 women who had no blood and/or urine test between 6 months to 3 years prepregnancy (Fig 1). Of the remaining 8,817 women, 247 (2.8 %) women had prepregnancy CKD, and the mean (SD) age was 30.2 (6.9) years. In total, 118 (47.7%) self-identified as Non-Hispanic African American, 91 (36.8%) self-identified as Hispanic, and 7 (2.8%) self-identified as non-Hispanic White (Table 1). With respect to CKD criteria thresholds, 179 (72.5%) had CKD modified G2 (eGFR 60-75 mL/min/1.73 m^2^ or urine protein or albumin to creatinine 201-500 mg/g), 27 (10.9%) had moderate CKD (eGFR 45-59 mL/min/1.73 m^2^ or urine protein or albumin to creatinine 501-1,000 mg/g), and 41 (16.2%) had severe CKD (eGFR <45 mL/min/1.73 m^2^ or urine protein or albumin to creatinine > 1,000 mg/g) (Fig 1).

**Table 1:** Baseline Characteristics by Prepregnancy eGFR (mL/min/1.73 m^2^) and Proteinuria Threshold Used to Define CKD (Urine Protein Creatinine Ratio, mg/g)

	Total Cohort N = 247	Prepregnancy 60 ≤ eGFR<75 mL/min/1.73 m^2^ or 200 < UPCR ≤ 500 mg/g N = 179	Prepregnancy eGFR 45 ≤ eGFR<60 mL/min/1.73 m^2^ or 500 < UPCR ≤1,000 mg/g N = 27	Prepregnancy eGFR <45 mL/min/1.73 m^2^ or UPCR >1,000 mg/g N = 41	*P* Value
Demographic parameters					
Mean age (y)	30.2 (SD 6.9)	30.4 (SD 7.0)	29.3 (SD 6.9)	30.0 (SD 6.9)	0.99
Race (n, %)					0.05
White	7 (2.8)	6 (3.3)	1 (3.7)	0	
Non-Hispanic African American	118 (47.7)	91 (50.8)	9 (33.3)	18 (43.9)	
Hispanic	91 (36.8)	60 (33.5)	16 (59.3)	15 (36.6)	
Asian	7 (2.8)	3 (1.7)	1 (3.7)	3 (7.3)	
Others	24 (9.7)	19 (10.6)	0	5 (12.2)	
Preferred language (n, %)					0.6
English	222 (89.8)	162 (73.0)	25 (11.3)	35 (15.8)	
Spanish	18 (7.2)	13 (72.2)	1 (5.6)	4 (22.2)	
Other	7 (3)	4 (57.2)	1 (14.3)	2 (28.6)	
Insurance (n, %)					0.1
Medicaid/managed Medicaid	148 (59.9)	105 (58.7)	17 (63.0)	26 (63.4)	
Commercial	58 (23.5)	45 (25.1)	6 (22.2)	7 (17.1)	
Medicare/managed Medicare	11 (4.5)	5 (2.8)	6 (3.7)	7 (12.2)	
Union	13 (5.2)	10 (5.6)	0 (0)	3 (7.3)	
Other	17 (6.9)	14 (7.8)	3 (11.1)	0	
Median SES (25-75 IQR)	–3.6 (SD 2.6)	–2.73 (–6.28 to 1.28)	–3.42 (–6.06 to 1.10)	–3.71 (–6.38 to 2.00)	0.6
Married or lives with significant other (n, %)	103 (41.7)	75 (41.9)	12 (44.4)	16 (39.0)	0.9
Comorbid conditions					
Median BMI	29 (IQR 25.1-35.5)	29.3 (25.2-35.9)	29.9 (25.8-35.9)	27.6 (24.1-33.6)	0.6
HTN (n,%)	101 (40.9)	64 (35.8)	11 (40.7)	26 (63.4)	0.04
Type of HTN					
Chronic HTN	79 (78.2)	45 (70.3)	10 (90.9)	24 (92.3)	
Gestational HTN	22 (21.8)	19 (29.7)	1 (9.1)	2 (7.7)	
DM (n,%)	51 (20.6)	32 (17.8)	7 (25.9)	12 (29.2)	0.04
Type of DM					
Type 2 DM	15 (29.4)	6 (17.6)	1 (14.2)	8 (66.6)	
Type 1 DM	3 (5.9)	2 (5.8)	0	1 (8.3)	
Gestational DM	33 (64.7)	24 (70.5)	6 (85.7)	3 (25)	
Rheumatological diseases (n, %)	10 (4)	1 (0.6)	2 (7.4)	7 (17.1)	<0.001
Sickle cell disease (n, %)	4 (1.6)	3 (1.7)	1 (3.7)	0	0.5
Antiphospholipid syndrome (n, %)	6 (2.4)	1 (0.6)	1 (3.7)	4 (9.8)	0.002
History of smoking (n, %)	24 (9.7)	19 (10.6)	2 (7.4)	3 (7.3)	0.7

HTN, hypertension.

The median number of days between cohort defining prepregnancy index clinic visit and pregnancy event was 340.5 (IQR, 178-675). The median number of days between prepregnancy eGFR measurement and pregnancy event was 424.5 days (IQR, 256-655) and between prepregnancy proteinuria measurement and pregnancy event was 570.5 days (IQR, 401-803).

The median body mass index was 29 (IQR, 25.1-35.5), and 103 (41.7%) were married or living with a significant other. With respect to health insurance, 148 (59.9%) were insured by Medicaid/managed Medicaid, and 58 (23.5%) had commercial insurance. The mean SES was –3.6 (SD 2.6) with the New York state mean represented as 0. With respect to comorbid conditions, 79 (78.2%) had chronic hypertension and 51 (20.6%) had diabetes. A history of rheumatological diseases, sickle cell disease, and antiphospholipid syndrome were present in 10 (4%), 4 (1.6%), and 6 (2.4%) of the women, respectively, whereas 24 (9.7 %) women reported a history of smoking. Restriction of the cohort to those with eGFR<45 mL/min/1.73 m^2^ and/or proteinuria>1,000 mg/g showed a higher prevalence of diabetes, hypertension, and rheumatologic disease (Table 1). With respect to pregnancy outcomes, 128 (51.8%) had a vaginal delivery, 72 (29.1%) had a cesarean section, 27 (10.9%) had a missed abortion, and 20 (8.1%) had a spontaneous abortion.

### Recognition of CKD and Peripregnancy Health Care Utilization

CKD was documented on the problem list in 32 out of 247 (12.9%) of women. When the sample was restricted to those with severe CKD, 21 out of 41(51.2%) had CKD documented in the problem list. Despite having prepregnancy CKD, only 68 out of 247 (27.6%) of women were seen by a primary care physician, and 20 out of 247 (0.1%) saw a nephrologist within 1 year before pregnancy event. Furthermore, 98 out of 247 (39.8%) of the total had primary care appointments arranged at discharge, whereas 23 out of 98 (23.5%) of these appointments were either canceled or not attended by patients. The mean time to primary care appointments was 18.1 weeks in the entire cohort and 20.6 weeks in those with severe CKD. Before pregnancy, 15 (36.5%) of women with severe CKD were seen by a nephrologist, and 20 (48.7%) had a nephrology appointment arranged at time of discharge. The median time to nephrology appointment in those with severe CKD was 21 weeks. RAASi was resumed in 45 out of 247 (18.2%) of the entire cohort, whereas the median time to start of RAASi was 471 days postpartum (Table 2). Of note, only 8 out of 247 (3.2%) of this cohort was accounted for by cohort entry between March of 2020 and January 1 2021, when the coronavirus disease (COVID) pandemic halted outpatient operations at MHS.

**Table 2:** Obstetric, Fetal, and Health Outcomes Among Women With Prepregnancy CKD in the Different Threshold Categories Used to Define CKD

	Total Cohort N = 247	Prepregnancy 60 ≤ eGFR < 75 mL/min/1.73 m^2^ or 200 < UPCR ≤500 mg/g N = 179	Prepregnancy eGFR 45 ≤ eGFR<60 mL/min/1.73 m^2^ or 500 < UPCR ≤1,000 mg/g N = 27	Prepregnancy eGFR < 45 mL/min/1.73 m^2^ or UPCR >1,000 mg/g N = 41	*P* Value^a^
Obstetric outcomes					
Type of pregnancy event (n,%)					0.2
Vaginal delivery	128 (51.8)	93 (52.0)	18 (66.7)	17 (41.4)	
C section	72 (29.1)	54 (30.2)	5 (18.5)	13 (31.7)	
Spontaneous abortion	20 (8.1)	12 (6.7)	1 (3.7)	7 (17.1)	
Missed abortion	27 (10.9)	20 (11.2)	3 (11.1)	4 (9.8)	
Median gestational age (wk; IQR)	38.9 (IQR 37-40) n = 200	38.5 (35.7-39.7)	38.4 (33.9-39.4)	33.9 (25.1-39.0)	0.2
Gestational age (n,%)	n = 200				<0.001
<28 wk	4 (2.0)	2 (1.4)	1 (4.3)	1 (3.3)	
28-34 wk	21 (10.5)	8 (4.5)	2 (7.4)	11 (26.8)	
>34 wk	175 (87.5)	137 (93.2)	20 (87.0)	18 (10.3)	
Gestational HTN (n, %)	101 (50.5)	16 (34.7)	16 (76.2)	69 (68.3)	0.002
Pre-eclampsia (n, %)	34 (17)	18 (10.1)	6 (22.3)	10 (24.4)	0.02
Fetal outcome					
Median baby weight (g)	3,125 (IQR 2,695-3,512) (n = 172)	3,172.5 (2,740-3,480)	3,155 (2,990-3,815)	2,595 (1,540-3,317)	0.005
Birth weight (n, %)	n = 172				0.003
<1,500 g	8 (4.6)	3 (2.4)	1 (5.0)	4 (14.8)	
1,500-2,500 g	25 (14.5)	16 (12.8)	1 (5.0)	8 (29.6)	
>2,500 gm	139 (80.8)	106 (84.8)	18 (90.0)	15 (55.6)	
NICU admission (n,%)	n = 200				0.001
Yes	40 (20)	22 (15.0)	4 (14.8)	14 (34.2)	
Unknown	7 (3.5)	4 (2.7)	1 (4.4)	2 (6.7)	
AKI during pregnancy (n,%)	14 (5.6)	2 (1.1)	2 (7.4)	10 (24.4)	<0.001
Health care utilization outcomes					
Mean length of stay (days)	2.6 (SD 0.9)	2.3 (1.1)	2.3 (1.2)	2.5 (1.7)	0.8
CKD included on problem list (n, %)	32 (12.9)	2 (1.1)	9 (33.3)	21 (51.2)	<0.001
NSAID prescription postpartum, up to 1 y after pregnancy (n, %)	234 (94.7)	174 (97.2)	27 (100)	33 (80.5)	<0.001
ACEI/ARB resumed postdischarge (n,%)	45 (18.2)	17 (9.5)	10 (37.0)	18 (43.9)	<0.001
Median time to ACEI/ARB resumed (d)	471 (IQ 116-1,624) n = 43	1,236 (467.5-1,648.5) n = 16	1,290 (350-2,021) n = 10	133 (4-344) n = 17	0.05
Seen by PCP within 1 y before pregnancy event (n, %)	68 (27.6)	44 (24.5)	5 (18.5)	19 (46.3)	0.05
PCP appt postdischarge within 1 year (n, %)	98 (39.8)	68 (37.9)	10 (37.0)	20 (50)	0.7
Seen by PCP with 1 year postpartum (n, % of those with appts made)	75 (30.4)	49 (27.3)	8 (80.0)	18 (90.0)	0.97
Canceled	5	5 (7.3)	0	0	
No show	18	14 (20.5)	2 (20.0)	2 (10.0)	
Weeks to PCP appt (mean, SD)	18.1 (14.9)	17.5 (14.6)	17.7 (20.6)	20.6 (14.2)	0.7
Seen by nephrology before pregnancy (n, %)	20 (0.08)	1 (0.6)	4 (14.8)	15 (36.5)	<0.001
Seen by nephrology during pregnancy (n, %)	21 (0.08)	3 (1.6)	5 (18.5)	13 (36.6)	<0.001
Dialysis	2			2 (4.9)	
Nephrology appt arranged postdischarge (n, %)	30 (0.12)	3 (1.7)	7 (25.9)	20 (48.9)	<0.001
Seen by renal post discharge (n, %)	29 (0.11)	4 (2.2)^a^	6 (85.7)	19 (95.0)	<0.001
Canceled/no show	1	0	1 (14.3)	0	
Dialysis	1	0	0	1 (5.0)	
Median time to renal appt post discharge (wk)	18 (IQ 6-62)	54 (22-150.5)	6 (3-7)	21 (9-62)	0.2

*Note:* Categories are not mutually exclusive.

ACEi, angiotensin-converting enzyme inhibitor; AKI, acute kidney injury; Appt, appointment; ARB, angiotensin receptor blocker; CKD, chronic kidney disease; eGFR, estimated glomerular filtration rate; HTN, hypertension; NICU, neonatal intensive care unit; NSAIDs, nonsteroidal anti-inflammatory drugs; PCP, primary care physician.

a One appointment made after discharge.

### Pregnancy and Fetal Outcomes

The median gestational age was 38.9 weeks among those pregnancies that reached viability, of which 25 (12.5 %) had early preterm deliveries, 101 (50.5 %) developed gestational hypertension, and 34 (17 %) developed pre-eclampsia (Table 2). The median infant weight was 3,125 gm and the incidence of LBW and very LBW infants in this cohort was 14.5 % and 4.6%, respectively, with 40 (20%) of infants requiring neonatal intensive care unit (NICU) admission (Table 2). When the cohort was restricted to those with severe CKD, a higher proportion had spontaneous abortions and preterm deliveries, with higher incidence of LBW and NICU admission and acute kidney injury (AKI) during pregnancy (Table 2).

### Association Between eGFR and Proteinuria Thresholds and CKD Trends Postpregnancy

**Figure 2:**
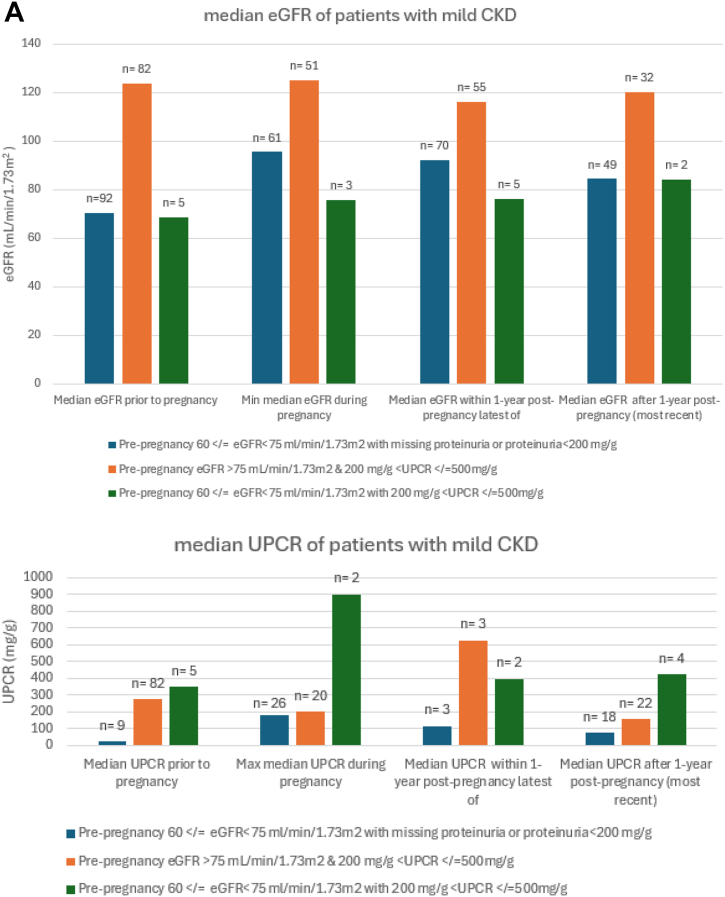

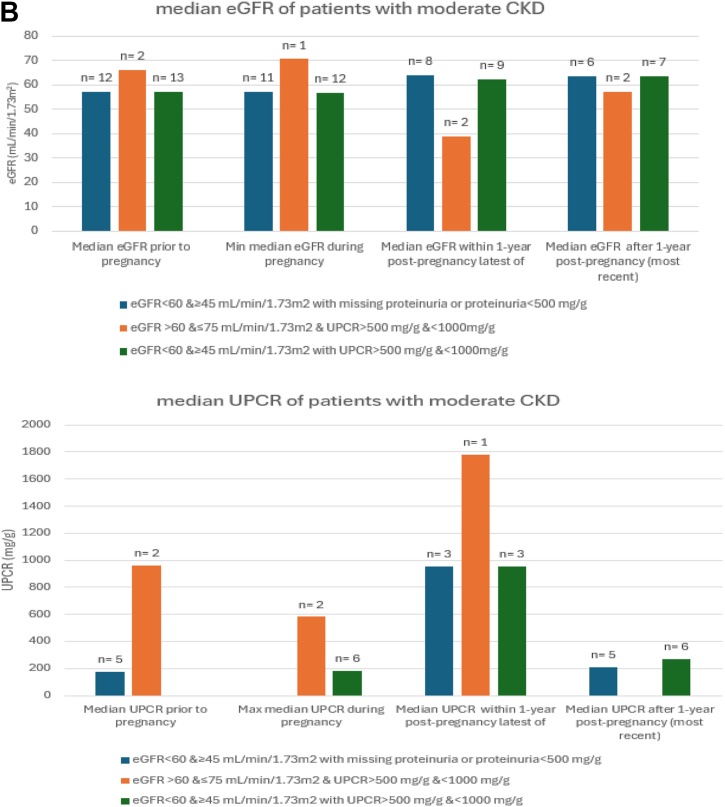

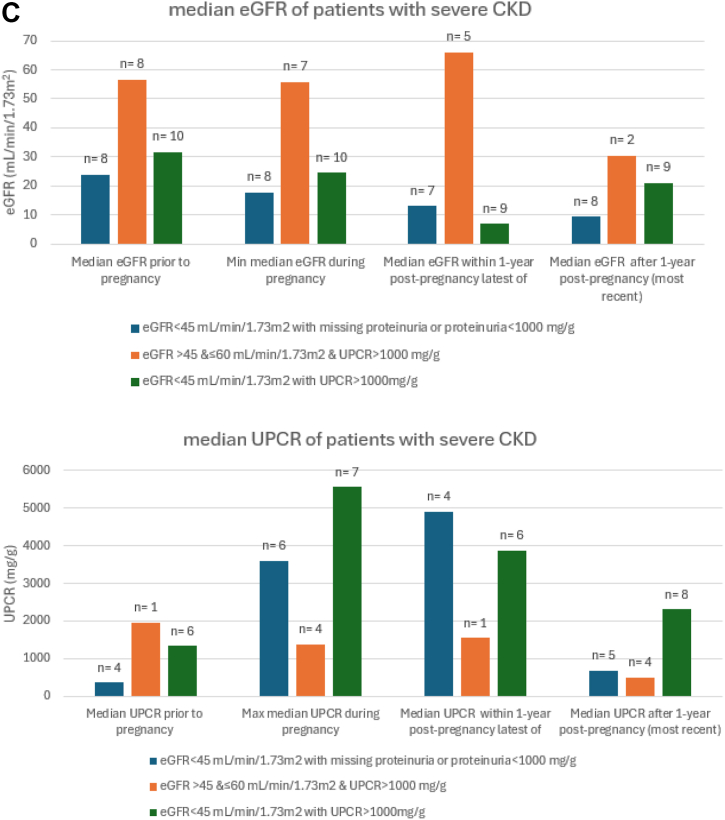
Patterns of eGFR and proteinuria during and after pregnancy stratified by increasing degree of prepregnancy kidney disease. (A) The modified KDIGO CKD stage G2 (60 ≤ eGFR < 75 mL/min/1.73 m^2^) and proteinuria ≤ 500 mg/g. (B) CKD G3a (eGFR <60 and ≥ 45 mL/min/1.73 m^2^ or proteinuria > 500 mg/g and <1,000 mg/g). (C) eGFR < 45 mL/min/1.73 m^2^ and proteinuria > 1,000 mg/g.

When we used the modified KDIGO CKD G2 (60 ≤ eGFR < 75 mL/min/1.73 m^2^) and proteinuria >200 and < 500 mg/g to identify CKD among prepregnant women, kidney function remained impaired postpregnancy. The median proteinuria peaked during pregnancy at 896 mg/g (384-1,407 mg/g) and remained high at 421 mg/g (IQR, 151-6957 mg/g) 1 year postpartum (Fig 2A). In the subgroup with eGFR <75 mL/min/1.73 m^2^ who had missing proteinuria or proteinuria < 200 mg/g, eGFR remained < 90 mL/min/1.73 m^2^ after 1 year post pregnancy. In the subgroup with eGFR>75 mL/min/1.73 m^2^ and proteinuria > 200 mg/g and < 500 mg/g, the median proteinuria peaked within 1 year postpregnancy at 623 mg/g (IQR, 4-1,202 mg/g) but improved to 154 mg/g (IQR, 58-479 mg/g) beyond 1 year postpregnancy. Although kidney impairment persisted after 1 year postpregnancy, it was most marked in those women who had both prepregnancy reduced eGFR and greater proteinuria (Fig 2).

In patients with moderate CKD (eGFR <60 and ≥ 45 mL/min/1.73 m^2^ or urine protein or albumin to creatinine >500 mg/g and <1,000 mg/g), there was persistence of kidney impairment postpregnancy (Fig 2B). The median proteinuria remained high within 1 year post pregnancy at 954 mg/g (468-1,545 mg/g) but improved at 1 year postpregnancy to 268 mg/g (IQR, 153-680 mg/g). Similarly, kidney function remained severely affected postpartum (Fig 2C) in those with severe CKD with median eGFR at 21 mL/min/1.73 m^2^ (IQR, 10-32) at 1 year postpregnancy, and median proteinuria worsening during pregnancy at 5,565 mg/g (IQR, 1250-8577 mg/g) and remained higher than prepregnancy postpartum (Fig 2C). Notably, of those who had eGFR < 45 mL/min/1.73 m^2^ and/or proteinuria >1,000 mg/g, only 25 out of 41 (60.9%) had eGFR checked, and only 17 out of 41 (41.4%) had proteinuria checked during pregnancy. Within 1 year postpregnancy, only 51.2 % (21/41) had eGFR, and 26.8% (11/41) had proteinuria checked.

## Discussion

The prevalence of CKD among pregnant women in the United States appears to be increasing. Over the past 10 years, the mean age of pregnancy has increased significantly from a mean of 26.6 years in 2016 to 27.5 years in 2023, which correlates with a higher likelihood of cardiometabolic risk factors.^29^^,^^30^ Our study showed a high prevalence of diabetes and hypertension in those with evidence for prepregnancy CKD, which are consistent with these statistics. Furthermore, as the number of women conceiving with pre-existing CKD increases, there is a subset of women who may develop AKI during pregnancy; as we demonstrated, adverse pregnancy outcomes and persistent or worsening post pregnancy kidney disease. Prior research indicates that adverse pregnancy outcomes are more prevalent in African American and Latina women compared with non-Latina White women, and African American women experience a maternal mortality rate of 69.9 per 100,000 compared with 26.6 per 100,000 among non-Latina White women.^31^ AKI and CKD during pregnancy is also more prevalent among African American, Latina, and Medicaid-insured women compared with their White counterparts.^31^ Postpregnancy, this demographic is also less likely to engage in follow-up care, receive RAASi, or be adequately prepared for the transition from CKD to kidney failure and kidney replacement therapy.^15^^,^^32^^,^^33^ Pregnancy offers a unique opportunity for women to be diagnosed with CKD and to engage with health care services. In this study, we show in the presence of laboratory evidence for CKD, the diagnoses was grossly underrecognized with only 12.9% having CKD included on their problem list during pregnancy. To validate our diagnostic approach of using laboratory data and urine tests, in exploratory analyses, we examined the association of different eGFR and proteinuria cutoffs with postpregnancy CKD trends. From these findings, we can infer that the modified KDIGO definition of CKD G2, with eGFR defined as 75 mL/min/1.73 m^2^, is a valid diagnostic threshold for identifying women with prepregnancy CKD at risk for adverse outcomes, as is proteinuria >200 mg/g either alone or in combination with eGFR decrease defined above, with the latter providing the most specific prediction. In the general population, patients with early CKD stages are often asymptomatic, underscreened, and underdiagnosed.^34^ During pregnancy, making the diagnosis of CKD is further complicated by pregnancy associated glomerular hyperfiltration on the one hand, which gives a false impression of normal kidney function, and a lack of appreciation of the risk of pre-eclampsia and/or AKI for CKD progression on the other.^35^, ^36^, ^37^, ^38^, ^39^ Furthermore, testing for proteinuria was only performed when pre-eclampsia is suspected.^40^, ^41^, ^42^, ^43^ Both the United States Preventive Services Task Force and American College of Obstetrics and Gynecology recommend against routine screening for pre-eclampsia using the urine dipstick for protein in low-risk women; however, they both recommend routine screening for hypertensive disorders in pregnant women with blood pressure measurements. Because young healthy women are seldom under the care of a physician, kidney disease and/or hypertension may go undetected, as can postpregnancy surveillance.

We showed that only a low percentage of the women with the most severe CKD defined by lowest eGFR category and highest proteinuria category were followed by nephrology during pregnancy, and only half were given a postdischarge appointment with the median time to a nephrology appointment being 21 weeks. Furthermore, a low proportion of women were started on RAASi postpartum. Early resumption of these medications in the postpartum period is associated with better cardiovascular and kidney outcomes.^44^^,^^45^ Limited clinical data suggest to avoid angiotensin-converting enzyme inhibitors or angiotensin II receptor blockers by breastfeeding mothers of preterm babies in the first few weeks postpartum to avoid profound neonatal hypotension.^44^, ^45^, ^46^, ^47^, ^48^, ^49^ In our cohort, which represents a sample of women already engaged in care by virtue of their attendance to a clinic visit before pregnancy as a cohort entry requirement, less than 50% of those with prepregnancy CKD had eGFR and proteinuria checked by a laboratory during pregnancy. Women considering pregnancy who have comorbid conditions associated with CKD development, at the very least, should have diagnostic tests to evaluate for the presence of CKD and should be integrated into multidisciplinary high-risk obstetrics clinics.

Other studies support our findings. It is recommended that all women have contact with their obstetric care providers within the first 3 weeks postpartum. This should be followed up with a more complete comprehensive postpartum visit no later than 12 weeks after birth by obstetrics or primary care.^50^ The current standard of care is a 6-week postpartum visit. These visits will provide an opportunity to identify patients with higher predicted cardiovascular morbidity and will help transition them to longitudinal care by a primary care provider and a specialist as needed.^51^, ^52^, ^53^ However, there is marked underutilization of these visits for poorly understood reasons.^50^^,^^51^^,^^53^^,^^54^ Gemkow et al^55^ found that less than 20% of 7,926 postpartum patients and only 25% of high-risk patients who received care at federally qualified health centers transitioned to primary care at 6 months postdelivery. In another study conducted on claims data in Maryland, approximately 50% of women attended a primary care visit postpartum.^53^ Among a nationwide sample of women with gestational diabetes mellitus, postpartum primary care visit completion was only 13%.^56^ In another study, of patients with pre-eclampsia and elevated postpartum blood pressure, only 12% received a referral to see a specialist.^57^ Barriers such as insurance shortfalls, inadequate knowledge of postpartum care, difficulty with accessing care because of transportation and competing priorities, scheduling challenges, and underutilization of mental health resources have been identified.^58^, ^59^, ^60^, ^61^ We showed shortfalls in arrangement of appointments and recognition as CKD as possible drivers of underutilization and disturbing gaps in health system efforts to transition to women to postpartum care.^50^^,^^51^^,^^53^

We suggest that health care systems better support postpregnancy health utilization through awareness campaigns, encouraging regular health maintenance visits between pregnancies, understanding social needs that act as barriers to regularly follow-up, and developing strategies to circumvent these barriers, including more frequent use of CKD testing and specialized nephrology consultations as well as artificial intelligence usage for early intervention and targeted health interventions.^62^^,^^63^ Involvement of community health workers, multidisciplinary OB-renal clinic, and well-run postpartum clinic are some of the other ways to identify and manage these high-risk patients.

Limitations of our study include its retrospective design, single-center data source, absence of a control group of pregnant women without CKD, and the introduction of sampling bias by only including women who had been engaged in care before pregnancy wherein they were at higher risk for adverse outcomes. Our sample size was not large enough to allow for multivariate regression modeling. We did not include individuals with eGFR levels above 75 mL/min/1.73 m^2^ because we prioritized specificity of our CKD definition over sensitivity to increase the inferential yield of the associations found. Strengths include minimization of sampling and ascertainment bias by defining a relevant denominator population based on those engaged with care in Bronx MHS from which to draw accurate estimates of incidence and prevalence, and a focus on women who self-identify as racial or ethnic minorities.

In conclusion, our study shows disturbing gaps in the diagnosis of CKD and utilization of pre and postpartum health services, among those with prepregnancy laboratory evidence for CKD. An eGFR of <75 mL/min/1.73 m^2^ and/or proteinuria > 200 mg/g is an acceptable threshold to identify high-risk women who would benefit from multidisciplinary care and intensive follow-up postpartum. With expansion of comprehensive insurance coverage after pregnancy through Medicaid and the Children’s Health Insurance Program for a full 12 months, it is crucial to understand barriers to health utilization and implement strategies to improve appropriate health care utilization among women with or at risk for CKD.
