# Supplementary material for: Health Care Utilization Among Pregnant Women With Pre-existing Kidney Disease: A Retrospective Cohort Analysis
Source: Kidney Med. 2025 Oct 14;7(12):101147. doi: 10.1016/j.xkme.2025.101147 (PMC12677166; doi:10.1016/j.xkme.2025.101147)
Supplement: Supplementary File (PDF) — Table S1. [file mmc1.pdf]

Type of outpatient visit at MHS 6months to 3 years pre-pregnancy	Number of women
Internal medicine/family medicine	63 (25.5%)
OBGYN	118 (47.7%)
Others	66 (26.7%)

Table S1: Type of outpatient visit for individuals who were engaged in care with MHS 6 months to 3 years pre-pregnancy
